# Can Natural Killer Cells Be a Principal Player in Anti-SARS-CoV-2 Immunity?

**DOI:** 10.3389/fimmu.2020.586765

**Published:** 2020-12-07

**Authors:** Faria Ahmed, Dong-Hyeon Jo, Seung-Hwan Lee

**Affiliations:** ^1^ Department of Nursing, Faculty of Health Sciences, University of Ottawa, Ottawa, ON, Canada; ^2^ Department of Biochemistry, Microbiology, and Immunology, Faculty of Medicine, University of Ottawa, Ottawa, ON, Canada; ^3^ The University of Ottawa Centre for Infection, Immunity, and Inflammation, Faculty of Medicine, University of Ottawa, Ottawa, ON, Canada

**Keywords:** natural killer cells, immunotherapy, immunomodulatory, COVID-19, SARS-CoV-2 infection

## Introduction

The spread of the coronavirus disease 2019 (COVID-19) pandemic has led to disastrous consequences, impacting social, economic, and medical systems, spanning both developed and developing countries (1). According to the World Health Organization, there have been over 35 million confirmed cases of COVID-19 at the time of writing, with over a million deaths worldwide (2). Within the larger Coronaviridae family, alpha-, beta-, gamma-, and delta-coronavirus genera are able to infect a variety of hosts (1). The beta coronaviruses (β-CoV) are notorious for having caused the Severe Acute Respiratory Syndrome (SARS) epidemic, the Middle East Respiratory Syndrome (MERS) epidemic, and now the COVID-19 pandemic. COVID-19 is caused by the SARS-CoV-2 virus, following the SARS-CoV and MERS-CoV, which caused the SARS and MERS epidemics, respectively (3–6).

Clinical manifestations of SARS-CoV-2 infection among symptomatic individuals can include cough, weakness, fever, and a spectrum of less common symptoms such as sore throat, rhinorrhea, hemoptysis, lymphopenia, and diarrhea (4, 7–10). Poorer outcomes are commonly observed among the elderly and individuals with co-morbidities (11–14). Those unable to clear the virus become critical and frequently develop Acute Respiratory Distress Syndrome (ARDS) (15–17). While immunological characteristics of COVID-19 continue to be discovered with ongoing research, the findings surrounding a cytokine storm as well as lymphopenia in patients with SARS-CoV-2, have been consistently reported in most studies (4, 11, 18–20). Elevated pro-inflammatory cytokines are associated with the severity of disease (13, 18, 20, 21).

Natural Killer (NK) cells play a central role in anti-viral immunity. They can lyse virus-infected host cells by inducing apoptosis *via* several different pathways. Primarily, it can release an array of proteins such as perforin and granzymes by exocytosis, and together these molecules can induce apoptosis of the target cell (22–24). Secondly, NK cells can kill the target by expressing FAS ligand (FasL) and TNF-related apoptosis-inducing ligand (TRAIL) as the executioner molecules, and subsequently initiate signaling down the extrinsic apoptotic pathway (24, 25). In addition, NK cells are able to secrete pro-inflammatory cytokines, such as IFN-γ, TNF-α, GM-CSF, as well as chemokines (25–28). NK cells show functional plasticity in the context of disease progression by eliciting pro-inflammatory or anti-inflammatory nature (29–32). Due to the reduced frequencies of NK cells found in COVID-19 patients (33–35) and the well-known anti-viral role of NK cells, it is tempting to speculate that the restoration of NK cells and their function would be a plausible solution for COVID-19 patients.

## Immune Characteristics of SARS-CoV-2 Infection

Postmortem biopsies of SARS-CoV-2 patients have revealed significant injury to alveolar epithelial cells and other signs of alveolar damage and inflammation (36). The target receptor through which SARS-CoV-2 (and SARS-CoV) enter host cells is the Angiotensin-Converting Enzyme 2 (ACE2) receptor; interestingly, this receptor is sparsely found on cells within healthy human lungs (3, 37, 38). However, several studies reported that the expression of ACE2 receptor in the lung tissue of individuals with respiratory conditions was increased (39–42). It would explain why the virus is successfully able to infect larger areas of lung tissue. The ACE2 receptor is not only limited to lung tissue but has also been identified in various other tissues in the body, including the cornea, gallbladder, heart, kidney, and testis, but it is most frequently distributed in nasal, alveolar, and intestinal epithelial cells (43, 44).

In a murine model of SARS-CoV-1 infection, increased production of chemokines was correlated with increased migration of various innate immune cells, including NK cells into the lungs (45). Various studies suggested chemokine-induced infiltration of immune cells in the infected lungs of patients (3, 46–48). Furthermore, the inflammatory response was followed by a second round of cytokine secretion several days later, and this is when T lymphocyte migration to lungs was subsequently observed (45). Other murine coronavirus infection models also reported the migration of NK cells to lung tissue (49). Thus, these patterns of immune cell migration to lungs observed among coronavirus infections allow for speculation regarding potential NK cell migration to lungs at the early stages of the SARS-CoV-2 infection as well. Under homeostatic conditions, human lung NK cells showed highly differentiated, but hyporesponsive phenotypes with continuous, dynamic movement between blood and the lung (50). Seemingly, the high expression of chemokines in the lungs of COVID-19 patients facilitates NK cell migration from the peripheral blood to the lungs. However, a recent single-cell RNAseq data of the major bronchoalveolar lavage fluid (BALF) immune cell types indicated that patients with severe/critical COVID-19 infection contained similar proportions of NK cells compared with healthy controls (51). Notably, NK cell numbers in peripheral blood mononuclear cells (PBMCs) were found significantly reduced in COVID-19 patients with severe disease compared to those with mild disease (52–54). Importantly, those NK cells showed increased expression of inhibitory receptor T-cell immunoglobulin and mucin domain-3 (TIM-3) (55). Thus, current evidence indicates a potential contraction of the NK cell population in the circulation of patients with severe SARS-CoV-2 infection (particularly pronounced with disease severity), even if there is no difference in NK cell counts within the lungs.

### Potential for NK Apoptosis in SARS-CoV-2 and Other Infections

Recurrent observation indicates that SARS-CoV-2 patients have lower total lymphocyte and total NK counts in circulation (33–35, 56–59). A similar reduction in NK cell counts had been previously reported for the SARS-CoV-1 infection (60, 61). In a small cohort of three patients, there was an increased expression of apoptotic genes among the PBMCs compared to healthy controls (21). It hints at increased lymphocyte apoptosis in a highly inflammatory milieu. Besides, current evidence supports that ratios of the various immune cell types are shifted during this infection. An increased neutrophil to lymphocyte ratio in the circulation of SARS-CoV-2 patients is associated with poor prognosis (62, 63). A case study of a patient with NK cell lymphoma, who was infected with SARS-CoV-2, revealed a reduction in NK clonal cell numbers as well as lymphoma load, putting them in remission; this was reversed once the patient had resolved the infection (64). This phenomenon raises interest in understanding the potential role of SARS-CoV-2 in inducing NK cell apoptosis.

A study performed single-cell sequencing of PBMC from patient groups with either SARS-CoV-2 or Influenza infection. Findings indicate increased levels of apoptotic molecules (XIAP associated factor 1, TNF, and FAS), leading to apoptosis of T cells in the COVID-19 patient group alone (52). Upcoming research should investigate if a similar activation of apoptotic molecules takes place in NK cells of severe and mildly infected SARS-CoV-2 patients, compared to naïve or other virally infected groups.

### The Dynamics of a Contracting and Expanding NK Population in Viral Infections

Past evidence demonstrated two contrasting outcomes in which NK cells are able to maintain or contract the size of their population in response to various viral infections (65–69). A severe reduction of CD56^dim^ NK cells, the major population mediating NK cell cytotoxicity, was observed in chronic human immunodeficiency virus (HIV), hepatitis C virus (HCV), and varicella-zoster virus (VZV) infections in humans (65). Interestingly, this severe, early loss of CD56dim is followed by an increase in CD3–CD56–CD161+ NK cells with low perforin and elevated SH2 domain-containing inositol 5-phosphatase-1 (SHIP-1) expression in chronic HIV-1 infection (70). It suggests that anergic phenotype is accompanied during the NK cell loss. In contrast, several reports demonstrated NK cell expansion in several viral infections in humans, including cytomegalovirus (CMV), chikungunya, Hepatitis B Virus (HBV), Epstein-Barr (EBV), and hantavirus infections (1, 3). In addition, the expanded population in early HIV infection particularly expressed increased levels of the activating receptor KIR3DS1 (71, 72).

Maintenance or expansion of the population during viral infections might be dependent on stimulation by a ligand-induced activating receptor, resulting in proliferation. Studies in mouse models of NK activating receptor Ly49H and m157 murine CMV (MCMV) glycoprotein have clearly demonstrated the importance of activating-receptor-induced activation to maintain the NK cell population during the prolonged MCMV infection (65, 73). Without the ligand-induced stimulation, NK cells seem markedly reduced in mice deficient in Ly49H during MCMV infection, similar to several viral infections.

During a typical viral infection, pro-inflammatory cytokines, such as Type 1 IFN, IL-12, IL-15, and IL-18, are generally elevated, inducing NK cell blastogenesis in early virus infection (74–76). Presumably, in a later stage following the innate cytokine-driven proliferation, persistent exposure of NK cells to the highly inflammatory milieu induces apoptosis; and stimulating signaling through the activating receptors might save NK cells from dying off by apoptosis. Thus, activating receptors on NK cells are not only required for the delivery of cytotoxicity and cytokine production, but are also required for the maintenance of the overall NK cell population in sustained virus infection. The model with MCMV also identified that the activating NK cell receptor endows NK cells to play a regulatory role. The sustaining NK cells showed a functional shift from pro-inflammatory to the anti-inflammatory state by producing IL-10. Without the ligand and activating receptor interaction, NK cells are lost during the prolonged infection and cannot take the opportunity to play the regulatory role, resulting in T cell-mediated immunopathology (73). Notably, NK cell loss or NK cell expansion with the functional switch is prominently observed on the stage, subsequent to early NK cell activation. Thus, the self-control circuit linking the presence of viral-specific NK cell receptors to the regulatory role seems ideal to limit collateral damage resulting from heightened immunity. It likely guarantees that NK cells receiving persistent stimulation *via* the receptors are programmed to change cytokine production profile to an anti-inflammatory orientation. Alternatively, in the absence of such activating receptors, NK cells are removed after their early IFN-γ production, thus preventing them from contributing to the cytokine storm.

While the mechanism by which NK cells are lost during SARS-CoV-2 virus infection remains elusive, recurrent observation of reduced NK cell frequencies and counts in COVID-19 patients might indicate the absence of ligand and activating receptor interaction between infected cells and NK cells. The NK cell loss is unlikely due to the infection-induced apoptosis in NK cells similar to that observed in the influenza virus infection (77) because NK cells do not express the host entry receptor ACE2 (78). It is tempting to speculate that NK cells in SARS-CoV-2 infection cannot recognize viral ligands of the highly mutated virus with pre-determined, germline-encoded, activating NK cell receptors, even though NK cells may kill the virus-infected cells by recognizing stress-inducible ligands.

A study that described detailed responses of NK cell to SARS-CoV-2 infection, showed that increased proportions of a subset of NK cell types called adaptive NK cells are found in severe but not moderate COVID-19 disease (55, 79). The absolute numbers of NK cells were lower in the blood of patients with COVID-19 compared with healthy controls, consistent with other reports. Adaptive NK cells are known to be generated by the epigenetic modification in several key signaling molecules upon CMV infection and sustained for life (80, 81). Interestingly, the adaptive NK cells possess an enhanced capacity for antibody-dependent cell-mediated cytotoxicity (ADCC) (80–83). While neutralizing antibodies against SARS-CoV-2 infected cells are the target of active investigation, understanding the role of the adaptive NK cells upon recognizing antibody-opsonized infected cells would provide comprehensive insight on NK cell function in COVID-19 patients.

### NK Cell Dysfunction in SARS-CoV-2 Infection

#### Inhibitory Pathways

Evidence indicates high expression of the immune checkpoint inhibitory receptor NKG2A on reduced NK cells in SARS-CoV-2 patients (84, 85). NKG2A prevents NK cell cytotoxicity by binding to the non-classical HLA-E molecule (86). A recent study has revealed that the SARS-CoV-2 Spike 1 protein can bind to the HLA-E and reduce NK cell degranulation function through the HLA-E/NKG2A pathway (87). Lung epithelial cells with intracellular expression of this Spike I protein *in vitro* resulted in an increased expression of HLA-E on its surface as well as an increase in expression of NKG2A (87). Gene expression of other inhibitory receptors such as TIM-3 and LAG-3 are also induced in NK cells from COVID-19 patients (88). Increased expression of those inhibitory receptors has been associated with immune exhaustion in chronic viral infections and may explain another mechanism by which the virus can override anti-viral immune responses (89).

#### Role of Complement

Numerous reports have reported significant similarities between the immune profile of COVID-19 and that of sepsis (90). In sepsis, the inflammatory protein C5a is generated from the cleavage of complement molecule C5 by the activation of C5 convertase (91). C5a can be generated *via* the action of alveolar macrophages and neutrophils, which have properties of the C5 convertase enzyme. One formed, C5a functions as an inflammatory mediator by binding a variety of immune cells expressing C5aR; it induces the production of various cytokines and chemokines in different cell types (91). Upon exposure to a sepsis-like immune environment, a subset of NK cells and NKT cells can display increased expression of C5aR receptors and increased cytokine production (92), resulting in detrimental roles of NKT and NK cells during early experimental sepsis. A recent study has demonstrated an increase in serum levels of soluble C5a among patients with SARS-CoV-2 infection with respect to disease severity (93). This leads us to speculate a potential role of C5a engagement with NK and NKT cells, making them notoriously contribute to cytokine storm. An ongoing clinical trial (NCT04371367) is investigating the potential reduction in hyperinflammatory response among COVID-19 patients with hypoxemic pneumonia using Avdoralimab, an antibody for C5a receptors.

#### Loss of Function

Consistently, SARS-CoV-2 patients reported reduced functional markers of NK cells with fewer NK cells being CD107a^+^ (a degranulation marker), IFN-γ^+^, IL-2^+^, and TNF-α^+^ among infected patients compared to healthy controls (85). They also found a lower expression of granzyme B in the NK cells of these patients. Thus, it would be interesting to investigate if the exhausted phenotype is similar to those with low perforin and elevated SHIP-1 expression in chronic HIV-1 infection (70). Furthermore, functional markers of NK cell cytotoxicity, such as perforin expression in NK cells, were found to be even lower among critical patients with intensive care unit (ICU) admission compared to non-ICU patients and healthy controls (94). Interestingly, recovered patients were found to have normal counts of NK and CD8^+^ T cells with reduced NKG2A levels (85), indicating potential reversal among recovered patients. Increased pro-inflammatory cytokines, such as IL-6 and IL-8, in the circulation of SARS-CoV-2 patients correlated with decreased cytotoxic functions of NK cells and CD8^+^ T cells (94). It is possible that the high levels of circulating IL-6 contribute to dampening the cytotoxic functions of these NK cells, as demonstrated previously (95, 96). As such, anti-IL-6 therapy could play a role in preventing some of the immune dysfunction observed in such patients (97). Antibodies against the IL-6 or IL-6 receptor, including siltuximab, tocilizumab, and sarilumab, are intensively being tested for therapeutics in COVID-19 patients in multiple settings (98, 99). Notably, a study in which five ICU patients received tocilizumab treatment, restored NK cells function, characterized by a significantly increased expression of granzyme A and perforin (35). In this study, an increased lymphocyte count was observed after tocilizumab treatment, but unfortunately, NK cell counts were not measured (35). Clinical trials with a larger number of patients can determine whether the treatment might correct the NK cell loss and dysfunction in COVID-19 patients.

Even though the counts of NK cells were reduced in patients with SARS-CoV-2 infection, they appeared to be activated (100). In particular, the NK cells from infected patients had increased expression of granzyme A and perforin, but a decreased expression of granzyme B compared to healthy controls. These findings collectively indicate the loss of some but not all functional characteristics of NK cells among SARS-CoV-2 patients.

## NK Cell-Mediated Immunotherapy in SARS-CoV-2 Infection

Due to the loss of NK cells in COVID-19 patients, it is straightforward to assume that the restoration of the number of NK cells by infusing *ex vivo* expanded NK cells into patients can reinstate immune capacity and increase chances of recovery. Numerous studies done previously for NK cell-mediated cancer therapies have already established various protocols for NK cell expansion from several sources, such as placental CD34+ cells, PBMC NK cells, or NK cell lines (101). Besides, NK cells are regarded as an ideal cell type for adoptive cell transfer due to the low risk of graft-versus-host disease (GVHD) (102). Thus, a few clinical trials are ongoing that attempt to infuse NK cells in COVID-19 patients to treat them (ClinicalTrials.gov Identifier: NCT04344548, NCT04365101, NCT04280224). A phase I-II trial is using an infusion of placental stem cell-derived CD34+ NK cells (that have been proliferated in tissue culture); they are aiming to treat symptomatic patients with mild to moderate SARS-CoV-2 infection and the trial will assess safety and efficacy of the therapy (NCT04365101). Another trial (NCT04280224) is using an infusion of NK cells twice a week on patients with SARS-CoV-2 infection who have developed pneumonia. It is unclear if the infusion contains primary NK cells from matched donors or are derived from other sources. The third study (NCT04344548) will be a Phase I–II trial performing adoptive cell transfer of allogenic, primary NK cells derived from healthy donors to COVID-19 patients with moderate to severe symptoms.

Considering the cytokine storm indicative of the highly inflammatory condition in COVID-19 patients, replenishing expanded NK cells should be designed with extreme caution. Although from healthy sources and expanded under controlled conditions, these NK cells will be exposed to the altered and pathogenic immune environment of these patients, with their unique cytokine milieu. This may cause the NK cell functions to become rather detrimental by producing a massive amount of cytokines and chemokines, instead of playing a beneficial role in the killing of infected cells. The MCMV model demonstrating a functional switch of sustained NK cells to anti-inflammatory might provide a hint for a plausible role of infused NK cells by calming the cytokine storm (73). During extended MCMV infection, NK cells are switched to produce the immunoregulatory cytokine IL-10. Mechanistically, the functional switch in NK cells is hard-wired to proliferation, guaranteeing that only sustained NK cells can achieve the intrinsic epigenetic changes in the *Il-10* gene locus (103). The proliferating NK cells acquire the histone modifications, allowing the chromatic structure of the *Il-10* locus from a closed to an open state. Thus, NK cells with the epigenetic modification can functionally switch to regulatory NK cells by producing IL-10 in response to various cytokine stimulations.

**Figure 1:**
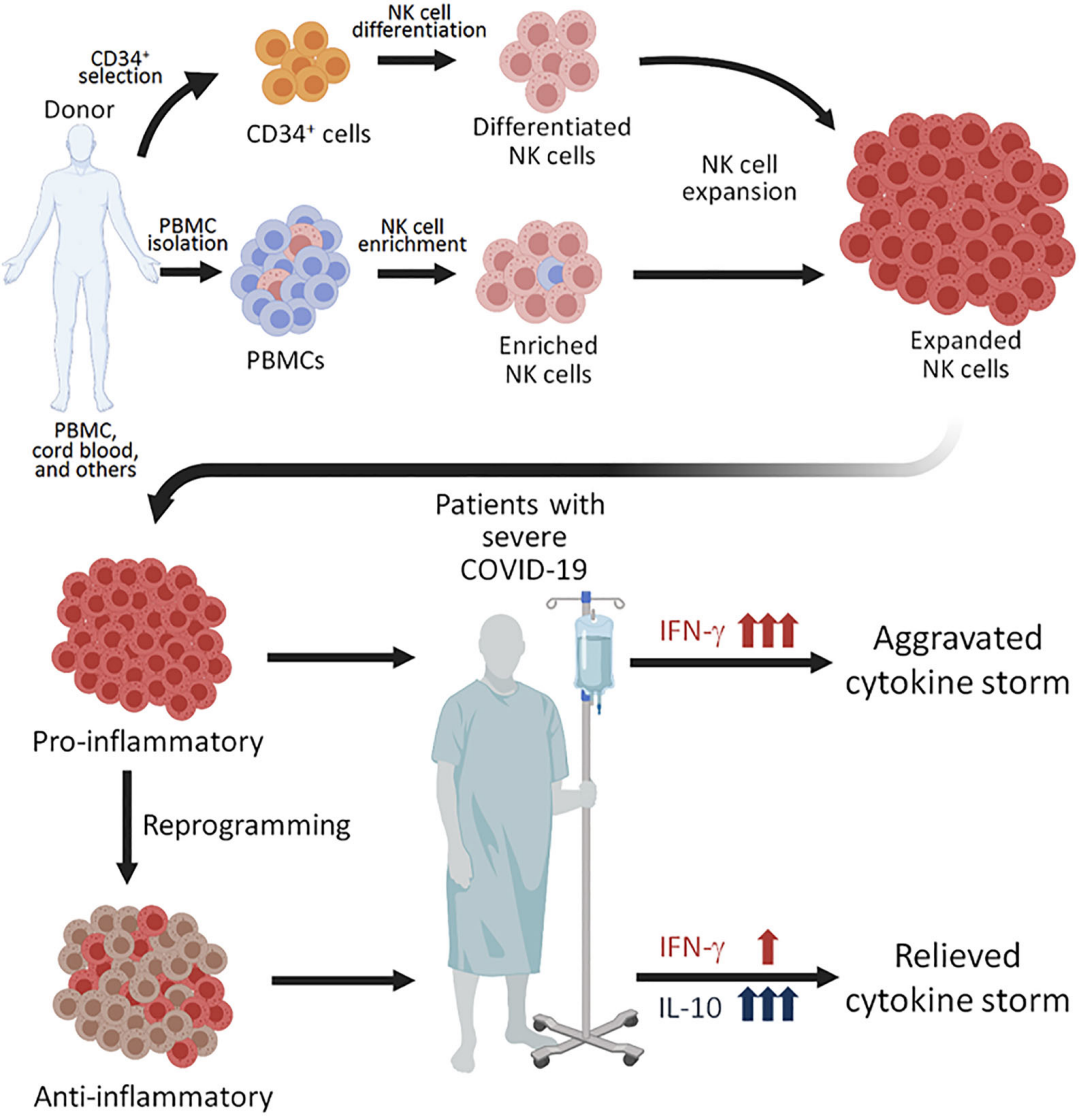
Proposed NK perfusion therapy to patients with severe COVID-19. NK cells can be obtained by CD56+ cell enrichment from healthy donor’s peripheral blood mononuclear cells (PBMCs), differentation of CD34+ hematopoietic progenitors from cord blood, and through other techniques. The NK cells can then be expanded using different combinations of cytokines and irradiated feeder cells. Since expanded NK cells are highly pro-inflammatory, perfusion of such NK cells to COVID-19 patients with severe disease. Alternatively, the expanded NK cells of pro-inflammatory NK cells. The NK cells reprogrammed to produced anti-inflammatory cytokine IL-10 may be more plausible for perfusion treatment for COVID-19 patients with severe disease, who are experiencing a cytokine storm.

Since *ex vivo* expanded NK cells have robustly divided during expansion by various protocols, the NK cells have presumably obtained the intrinsic epigenetic changes in the *Il-10* gene locus and might be able to respond to several cytokines that are known to induce IL-10 production from NK cells. For example, IL-12 can induce NK cells to produce IL-10 during inflammation and parasitic infection (104, 105), and IL-21 can also enhance IL-10 production by NK cell *in vitro* (106). Interestingly, IL-21 treatment on murine NK cells increases both cytotoxicity and IL-10 production (106), suggesting a treatment inducing IL-10 without compromising killing capacity of human NK cells. Therefore, pretreatment of expanded NK cells with cytokines to enhance IL-10 response might be an innovative strategy to tailor NK cell response and reduce immunopathology resulting from the excessive production of cytokines in COVID-19 patients (**Figure 1**).

## Concluding Remarks

The unprecedented impact of COVID-19 on social, economic, and medical systems urgently requires a deeper understanding of the immune responses associated with SARS-CoV-2 infection, to generate therapeutic responses. Although we remain in the early phases of filling this knowledge gap, some unique characteristics of the disease have begun to be consistently identified. These include the hyperinflammatory response and cytokine storm associated with the disease as well as lower counts of particular immune cells such as NK cells. Nonetheless, it may be naïve to assume that simply restoring NK cell numbers among COVID-19 patients will be sufficient to control the infection. The supplementary NK cells with pro-inflammatory properties may exaggerate the highly vulnerable situation in COVID-19 patients. Taking advantage of NK cell plasticity, we propose to employ NK cells that are reprogrammed to produce anti-inflammatory cytokine IL-10 in NK cell-mediated therapy. Alternately, NK cells can be reinvigorated by targeting the NKG2A/HLA-E inhibitory pathway. Overall, the review suggests that it is essential to investigate the mechanisms underlying NK cell losses to understand SARS-CoV-2 infection better and to design innovative immunotherapy for COVID-19 patients.

## Author Contributions

The review was prepared by the discussion and the consensus of all authors listed. FA and S-HL wrote the manuscript. All authors contributed to the article and approved the submitted version.

## Funding

This work was supported by funding from the Canadian Institutes of Health Research (PJT-156106) to S-HL. The University of Ottawa covered 50% of the article processing charge.

## Conflict of Interest

The authors declare that the research was conducted in the absence of any commercial or financial relationships that could be construed as a potential conflict of interest.
